# Different Roles of GRP78 on Cell Proliferation and Apoptosis in Cartilage Development

**DOI:** 10.3390/ijms160921153

**Published:** 2015-09-07

**Authors:** Zhangyuan Xiong, Rong Jiang, Xiangzhu Li, Yanna Liu, Fengjin Guo

**Affiliations:** 1 Department of Cell Biology and Genetics, Core Facility of Development Biology, Chongqing Medical University, Chongqing 400016, China; E-Mails: zyxiong38@sina.com (Z.X.); xzh11@yahoo.com (X.L.); yanlii@126.com (Y.L.); 2 Laboratory of Stem Cells and Tissue Engineering, Chongqing Medical University, Chongqing 400016, China; E-Mail: Rongjiang86@sohu.com

**Keywords:** GRP78, ER stress, apoptosis, unfolded protein response, cartilage development

## Abstract

Eukaryotic cells possess several mechanisms to adapt to endoplasmic reticulum (ER) stress and thereby survive. ER stress activates a set of signaling pathways collectively termed as the unfolded protein response (UPR). We previously reported that Bone morphogenetic protein 2 (BMP2) mediates mild ER stress and activates UPR signal molecules in chondrogenesis. The mammalian UPR protects the cell against the stress of misfolded proteins in the endoplasmic reticulum. Failure to adapt to ER stress causes the UPR to trigger apoptosis. Glucose regulated protein 78 (GRP78), as an important molecular chaperone in UPR signaling pathways, is responsible for binding to misfolded or unfolded protein during ER stress. However the influence on GRP78 in BMP2-induced chondrocyte differentiation has not yet been elucidated and the molecular mechanism underlyng these processes remain unexplored. Herein we demonstrate that overexpression of GRP78 enhanced cell proliferation in chondrocyte development with G1 phase advance, S phase increasing and G2-M phase transition. Furthermore, overexpression of GRP78 inhibited ER stress-mediated apoptosis and then reduced apoptosis in chondrogenesis induced by BMP2, as assayed by cleaved caspase3, caspase12, C/EBP homologous protein (CHOP/DDIT3/GADD153), p-JNK (phosphorylated c-Jun N-terminal kinase) expression during the course of chondrocyte differentiation by Western blot. In addition, flow cytometry (FCM) assay, terminal deoxynucleotidyl transferase-mediated deoxyuridine triphosphate-biotin nick end-labeling (TUNEL) assay and immune-histochemistry analysis also proved this result *in vitro* and *in vivo*. It was demonstrated that GRP78 knockdown via siRNA activated the ER stress-specific caspase cascade in developing chondrocyte tissue. Collectively, these findings reveal a novel critical role of GRP78 in regulating ER stress-mediated apoptosis in cartilage development and the molecular mechanisms involved.

## 1. Introduction

During embryonic development, chondrogenesis plays a fundamental role in skeletal patterning, bone formation, and joint development. Well-orchestrated chondrogenesis is controlled exquisitely by cellular interactions with the cytokines, growth factors, surrounding matrix proteins, and other environmental factors that mediate cellular signaling pathways and transcription of specific genes in a temporal-spatial manner [1,2,3]. Bone morphogenetic proteins (BMPs) are important cytokines and play several important roles in a variety of cellular functions ranging from embryogenesis, cell growth, and differentiation to bone development and the repair of bone fractures. BMP2, as a member of BMPs, is the most important regulators of cartilage development and bone formation [4,5]. Accumulated evidence indicates a physiological role of unfolded protein response of ER stress during developmental processes. BMP2 was reported to activate UPR signaling molecules, such as binding immunoglobulin protein (BiP), inositol-requiring enzyme-1α (IRE1α), old astrocyte specifically induced substance (OASIS), also named CREB3L1, activating transcription factor 6 (ATF6) and PKR-like ER-resistant kinase (PERK) [6,7,8]. Murakami *et al.* [9] reported that another BMP2 signaling pathway in osteoblasts was mediated by the UPR of ER stress and the expression levels of the ER stress markers, such as BiP, CHOP (C/EBP homologous protein) and ATF4 (activating transcription factor 4), were upregulated by BMP2 stimulation.

UPR, as a set of signaling pathways activated by ER stress, is primarily a response to relieve ER stress and promotes cell survival by improving the balance between the protein load and the folding capacity in the ER and/or by improving the secretion of trophic factors/growth factors. If the protein loaded in the ER exceeds its folding capacity, or some defects in the UPR exist, the cells are destroyed by apoptosis. Growing evidence has shown that excessively strong and lengthy ER stress will result in apoptosis. This is called ER stress-induced cell death [10,11,12].

GRP78, also referred to as BiP, is a central regulator of ER function due to its roles in protein folding and assembly, targeting misfolded protein for degradation, ER Ca^2+^-binding and controlling the activation of trans-membrane ER stress sensors [13,14,15]. We previously reported that ER stress is induced during BMP2-mediated chondrocyte differentiation and activates the IRE1α-XBP1 pathway. The interaction and dissociation between BiP and IRE1α are connected with chondrocyte physiological condition. BiP can interact with IRE1α in unstressed cells and dissociate from IRE1α in BMP2-induced condition. XBP1S positively regulates endochondral bone formation by activating granulin-epithelin precursor (GEP) chondrogenic growth factor [16,17]. However, the role of GRP78 in the ER stress-mediated apoptosis in cartilage development is poorly understood. Specifically, whether and how GRP78 influences the apoptosis in chondrocyte differentiation and the molecular mechanism underlying these processes remained unexplored. In the current study, we attempt to clarify the impact of GRP78 in ER stress-mediated apoptosis during the course of chondrogenesis, with a special focus on associated molecules of ER stress-mediated apoptosis in cartilage development, and the molecular events in this process.

## 2. Results

### 2.1. Identification of the Expression of Ad-GRP78 and Ad-siGRP78

**Figure 1:**
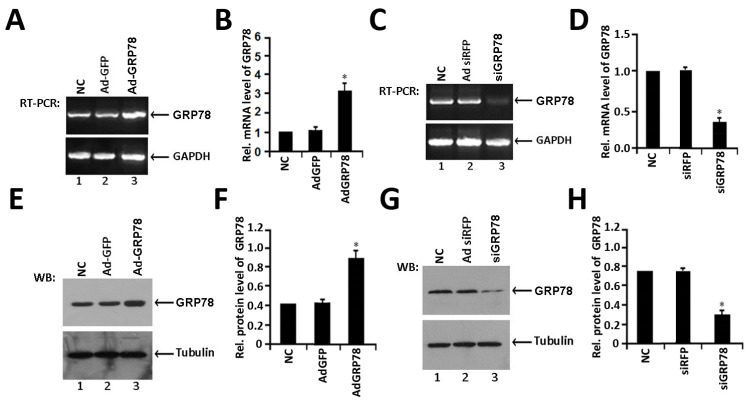
Expression of GRP78 in C3H10T1/2 cells after infected with Ad-GRP78 or Ad-siGRP78. (**A)**. Analysis of GRP78 mRNA level with RT-PCR. *Lane 1*: NC; *lane 2*: Ad-GFP control; *lane 3*: Ad-GRP78. C3H10T1/2 cells infected with Ad-GRP78 and control Ad-GFP, NC group were cultured for 48 h and GRP78 mRNA expression was determined by real-time PCR; (**B**). Quantification of relative levels of GRP78 in C3H10T1/2 cells. The normalized values were then calibrated against the control value, data were expressed as means ± S.D. (*n* = 3). The left bar indicates a relative level of GRP78 mRNA of 1; * *p* < 0.05; (**C**) Analysis of GRP78 mRNA level with RT-PCR. *Lane 1*: NC; *lane 2*: Ad-siRFP control; *lane 3*: Ad-siGRP78. C3H10T1/2 cells infected with Ad-siGRP78 and control Ad-siRFP, NC group were cultured for 48 h and GRP78 mRNA expression was determined by RT-PCR; (**D**) Quantification of relative levels of GRP78 in C3H10T1/2 cells. The normalized values were then calibrated against the control values, data were expressed as means ± S.D. (*n* = 3). The left bar indicates a relative level of GRP78 mRNA of 1; * *p* < 0.05; (**E**) Determination of GRP78 protein expression level after infected with Ad-GRP78. *Lane 1*: NC; *lane 2*: Ad-GFP control group; *lane 3*: Ad-GRP78 group. Proteins were separated by 10% SDS-PAGE and analyzed with anti-GRP78 antibody. Tubulin was used as internal control in Western blot. The levels of GRP78 proteins were obviously increased after infected with Ad-GRP78 compared with the control groups; (**F**) Semiquantification of relative levels of GRP78 in C3H10T1/2 cells. Levels were normalized against those of Tubulin by MJ Opticon Monitor Analysis Software (Bio-Rad, Hercules, CA, USA), data were expressed as means ± S.D. (*n* = 3). Every treatment group was compared with control groups respectively, * *p* < 0.05. Error bars, S.D.; (**G**) Determination of GRP78 protein expression level after infected with Ad-siGRP78. *Lane 1*: NC; *lane 2*: adenovirus si-GRP78 control group Ad-RFP; *lane 3*: Ad-siGRP78 group. Proteins were separated by 10% SDS-PAGE and analyzed with anti-GRP78 antibody. Tubulin was used as internal control in Western blot. The levels of GRP78 proteins were obviously reduced after infected with Ad-siGRP78 compared with the control groups; (**H**) Semi-quantification of relative levels of GRP78 in C3H10T1/2 cells. Levels were normalized against those of Tubulin by MJ Opticon Monitor Analysis Software (Bio-Rad), data were expressed as means ± S.D. (*n* = 3). Every treatment group was compared with control groups respectively, * *p* < 0.05. Error bars, S.D.

Ad-GRP78 and Ad-siGRP78 Adenoviruses vectors were constructed and identified with endonuclease digesting and DNA sequencing, respectively. The DNA-sequencing results indicated identical nucleotide sequence with the design (data not shown), which confirmed the correct construction of plasmids. Then the C3H10T1/2 cells infected with Ad-GRP78 were identified by RT-PCR and Western blot. The level of GRP78 mRNA obviously increased comparing with controls (Figure 1A,B). And protein levels were also significantly enhanced in Ad-GRP78 infected cells, comparing with the other two control cells, respectively (Figure 1E,F). Besides, as revealed in Figure 1C,D, the expression of GRP78 mRNA obviously decreased in Ad-siGRP78 infected cells comparing with controls. The protein levels were significantly reduced in Ad-siGRP78 infected cells, comparing with the other two control cells, respectively (Figure 1G,H). The results illustrated that the construction and expression of Ad-GRP78 and Ad-siGRP78 were correct.

### 2.2. Differential Expression of GRP78 in the Chondrogenesis of Micromass Culture of C3H10T1/2 Cells and ATDC5 Cells

**Figure 2:**
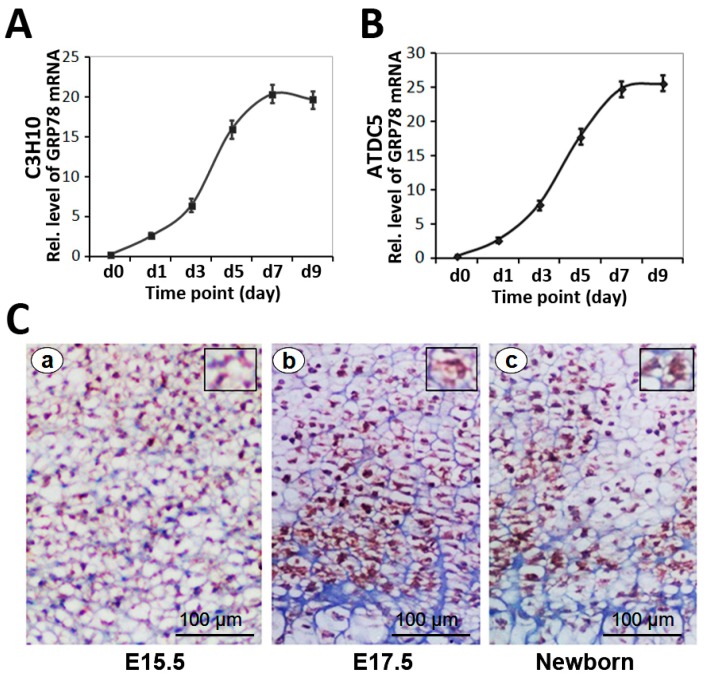
Expression of GRP78 during chondrogenesis both *in vitro* and *in vivo*. (**A**) and (**B**). Expressions of GRP78 was examined in the course of chondrogenesis of a micromass culture of C3H10T1/2 cells (**A**) and ATDC5 cells; (**B**) Micromass cultures of C3H10T1/2 cells were stimulated by BMP2 protein at various time points, as indicated, and the mRNA levels of GRP78 were assayed using real-time PCR. Units are arbitrary; the normalized values were calibrated against the d0 time point, here given the value of 1; (**C**) Immunohistochemistry of GRP78 in tibial growth plates of postcoital day 15.5 mouse embryo (E15.5; **a**), postcoital day 17.5 mouse embryo (E17.5; **b**), and newborn (**c**) is shown. Microphotographs are shown of sections stained with anti-GRP78 antibody (brown) and counterstained with hematoxylin (blue). Immunostaining reveals positive nuclear staining in the entire chondrogenic developmental stages in both proliferating and hypertrophic zones. Bar = 100 μm.

To deeply investigate GRP78 function in chondrogenesis, we first studied GRP78 expression profiles during chondrocyte differentiation in micromass culture of C3H10T1/2 cells and ATDC5 cells [17,18]. Micromass cultures of these cells were incubated in the presence of 300 ng/mL recombinant BMP2 for induction of chondrocyte differentiation. Cells were harvested at various time points followed by real-time PCR for measurements of GRP78 (Figure 2A,B). As shown in Figure 2A, the level of GRP78 was relatively low until day five, when it is doubled and thereafter remained at high levels during the differential stage in C3H10T1/2 cells. In addition, similar results were also observed in the course of chondrogenesis of ATDC5 cells (Figure 2B). Note that GRP78 was expressed in the entire process of chondrogenesis.

### 2.3. Expression of GRP78 in Growth Plate Chondrocytes in Vivo

We then examined the expression of GRP78 in growth plate chondrocytes using immunohistochemistry on tibial growth plates of mouse embryos on postcoital days 15.5, 17.5 and in the newborn. It is known that GRP78 acts as a major ER chaperone and a master regulator of ER stress signaling. As revealed in Figure 2C, GRP78 is detected at postcoital days E15.5, 17.5 and in the newborn, respectively. It was observed that GRP78 is expressed in the lumen of the endoplasmic reticulum (ER) in cytoplasm. In addition, GRP78 is observed in the entire growth plate of chondrocytes, including the proliferating zone and hypertrophic zone. The results demonstrated that GRP78 prominent expression throughout the growth plate chondrocytes is detected at postcoital days 15.5 and 17.5 and in the newborn. These results suggested that the expression profile of GRP78 is closely linked to the entire chondrogenesis stage.

### 2.4. Ad-GRP78 Reduces the Expression of XBP1S and Inhibits Kinetics of IRE1α Signaling in BMP2-Induced Chondrogenesis

**Figure 3:**
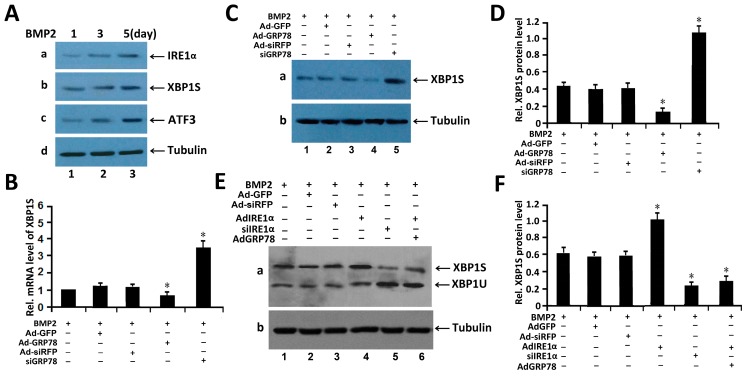
GRP78 decreases the expression of the XBP1S in BMP2-induced chondrogenesis. (**A**) Western blotting assay of the expression of ER stress associated molecules in C3H10T1/2 cells induced by BMP2 (300 ng/mL) for different times (1, 3, 5 days). Cell lysates prepared from micromass culture of C3H10T1/2 cells induced by BMP2, as indicated, were subjected to SDS-PAGE and detected with anti-BiP, anti-XBP1S, anti-IRE1α and anti-tubulin (serving as an internal control), respectively. Protein levels of IRE1α, XBP1S, ATF3 and tubulin (internal control) are shown; (**B**) Ad-GRP78 reduces, while siGRP78 increases, the level of XBP1S mRNA. C3H10T1/2 cells infected with Ad-GRP78 and control Ad-GFP, siGRP78 and control siRFP, were cultured for 48 h and endogenous XBP1S gene expression was determined by real-time PCR. The normalized values were then calibrated against the control value. The units are arbitrary, and the left bar indicates a relative level of XBP1S mRNA of 1; * *p* < 0.05; (**C**) Ad-GRP78 reduces, while siGRP78 increases, the level of XBP1S protein level in C3H10T1/2 cells induced by BMP2. C3H10T1/2 cells infected with Ad-GRP78 and control Ad-GFP, siGRP78 and control siRFP, were cultured for 48 h, respectively, and the endogenous XBP1S protein level was determined by Western blotting. siGRP78, an siRNA adenovirus targeting GRP78. Tubulin protein served as an internal control; (**D**) Semi-quantification of relative levels of XBP1S in micromass culture of C3H10T1/2 cells induced by BMP2. Levels were normalized against those of tubulin by MJ Opticon Monitor Analysis Software (Bio-Rad), data were expressed as means ± S.D. (*n* = 3). Every treatment group was compared with control groups respectively, * *p* < 0.05; (**E**) Ad-GRP78 decreases the level of XBP1S protein spliced by IRE1α in chondrocytes induced by BMP2. Micromass cultures of C3H10T1/2 cells were treated with 300 ng/mL BMP2, then C3H10T1/2 cells were infected with either Ad-GFP (serving as a control), siRFP (serving as a control), or Ad-IRE1α, siIRE1α, or Ad-IRE1α + Ad-GRP78, as indicated. Five or six days later, the cell lysates were used to detect the protein level of XBP1S by Western blotting. siIRE1α, an siRNA adenoviruse targeting IRE1α. Tubulin protein served as an internal control; (**F**) Semi-quantification of relative levels of XBP1S in micromass culture of C3H10T1/2 cells induced by BMP2. Levels were normalized against those of Tubulin by MJ Opticon Monitor Analysis Software (Bio-Rad), data were expressed as means ± S.D. (*n* = 3). Every treatment group was compared with control groups respectively, * *p* < 0.05. Error bars, S.D.

BMP2 is known to activate the unfolded protein response of ER stress [5,6,7]. We next did Western blotting to examine the expression profiling of UPR signaling molecules in BMP2-induced chondrocyte differentiation. For this purpose, Western blot analysis was performed in micromass culture of C3H10T1/2 cells with 300 ng/mL BMP2. The result showed that ER stress-associated molecules, such as IRE1α, XBP1S and ATF3, were activated and expressed in BMP2-treated cells (Figure 3A). Interestingly, with the BMP2 stimulation, the expression of IRE1α, XBP1S and ATF3 was also increased. The result showed that UPR signal pathway of ER stress has been activated after treatment with BMP2.

We next did real-time PCR assay and Western blotting with C3H10T1/2 cells. As revealed in Figure 3B,C, 48 h after infection, Ad-GRP78 remarkably decreased the XBP1S mRNA level, while siGRP78 clearly increased the mRNA level of XBP1S. These results were also verified by Western blotting at the protein level, as shown in Figure 3C. It is apparent that after infection with Ad-siGRP78 in ATDC5 cells induced by BMP2, XBP1S expression was enhanced, and overexpression of GRP78 can reduce XBP1S expression in ATDC5 cells induced by BMP2.

These data clearly indicate that GRP78 is able to regulate endogenous XBP1S gene expression. Micromass cultures of these cells were incubated in the presence of 300 ng/mL BMP2 for induction of chondrocyte differentiation and were infected with control Ad-GFP, siRFP, Ad-GRP78, Ad-IRE1α and siIRE1α. As revealed in Figure 3D, BMP2 induced ER stress, then Ad-IRE1α remarkably increased the XBP1S protein level. Ad-IRE1α obviously increased the XBP1S expression, while inhibition of IRE1α via the siRNA approach can reduce XBP1S expression and enhance XBP1U expression in BMP2-induced chondrogenesis comparing with siRFP control. In addition, overexpression of GRP78 can reduce the expression of IRE1α-spliced XBP1S and BMP2-induced IRE1α kinetic activity in chondrocytes differentiation. These data clearly indicate that IRE1α and GRP78 are able to regulate endogenous XBP1 gene expression and XBP1S splicing in chondrogenesis.

### 2.5. Impact of Ad-GRP78 and Ad-siGRP78 on Cell Growth in Chondrocyte Differentiation

**Figure 4:**
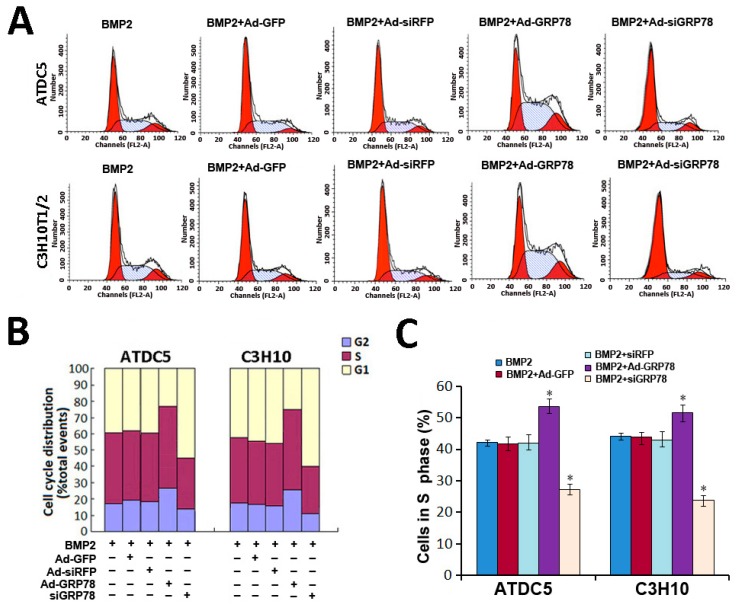
Cellular proliferation analysis by FCM. (**A**) Flow cytometry images with propidium iodide staining and analysis on cell cycle distribution. Micromass culture of ATDC5 cells and C3H10T1/2 were treated with BMP2 (300 ng/mL)/BMP2 + Ad-GFP/BMP2 + Ad-siRFP / BMP2 + Ad-GRP78 / BMP2 + Ad-siGRP78. Flow cytometry analysis showed that the percentage of the BMP2 + Ad-GRP78 ATDC5 cells in S phase were increased significantly compared to those in BMP2 controls, whereas the percentage of the BMP2 + Ad-siGRP78 ATDC5 cells in S phase were dramatically decreased compared with BMP2 control. The result of C3H10T1/2 is the same. Experiments were repeated three times, and samples were analyzed by Student’s *t*-test and statistical significance with *p* < 0.05. Representative images were shown; (**B**) Flow cytometry assay on the percentages of the ATDC5 and C3H10T1/2 cells in G2/M phase after treatment with BMP2 (300 ng/mL)/BMP2 + Ad-GFP / BMP2 + Ad-siRFP / BMP2 + Ad-GRP78 / BMP2 + Ad-siGRP78. * *p* < 0.05 compared with control; (**C**) Flow cytometry analysis showed that the percentages of the ATDC5 and C3H10T1/2 Ad-GRP78 cells in S phase were increased significantly, whereas the percentages of the ATDC5 and C3H10T1/2 Ad-siGRP78 cells in S phase were decreased compared with those in their controls. * *p* < 0.05 compared with control.

To investigate whether GRP78 can influence the cell cycle profile in chondrogenesis, flow cytometry analysis was undertaken to determine the effect of Ad-GRP78 and Ad-siGRP78 on cell cycle in BMP2-induced ATDC5 cells and C3H10T1/2 cells. As shown in Figure 4A,C, the data showed that the cell number in S phase was reduced in BMP2 + Ad-siGRP78 micromass culture of ATDC5 cells comparing with BMP2 and BMP2 + siRFP control. The cell number in S phase was 27.82% in BMP2 + Ad-siGRP78 ATDC5 cells and 23.67% in BMP2 + Ad-siGRP78 C3H10T1/2 cells. However, the cell number in S phase was increased in BMP2 + Ad-GRP78 micromass culture of ATDC5 cells comparing with BMP2 and BMP2 + AdGFP control. The cell number in S phase was 54.72% in BMP2 + Ad-GRP78 ATDC5 cells and 51.09% in BMP2 + Ad-GRP78 C3H10T1/2 cells. The differences between treatment groups and control groups have statistical significance (*p* < 0.05). These data indicate that GRP78 can influence cell cycle distribution. Overexpression of GRP78 enhances the cell number in S phase, while GRP78 knockdown decreases the cell number in S phase in chondrocyte differentiation. In addition, the data also showed that in ATDC5 cells, the percentage of G2 phase was reduced in BMP2 + Ad-siGRP78 group (14.78% ± 0.78%) compared with BMP2 + siRFP control and BMP2 group, while increased in BMP2 + Ad-GRP78 group (26.21% ± 0.81%) compared with BMP2 + AdGFP control and BMP2 group. In addition, in micromass culture of C3H10T1/2 cells, the percentage of G2 phase was reduced in BMP2+Ad-siGRP78 group (12.27% ± 0.62%) compared with BMP2 + siRFP control and BMP2 group, while increased in BMP2 + Ad-GRP78 group (27.08% ± 0.92%) compared with BMP2 + AdGFP control and BMP2 group (Figure 4B). The differences between treatment groups and control groups have statistical significance (*p* < 0.05). These data indicate that GRP78 can influence cell cycle distribution in chondrocyte differentiation. Overexpression of GRP78 enhanced, while GRP78 knockdown inhibited, the S phase cells in chondrogenesis.

These results suggested that overexpressed GRP78 can augment cell proliferation in chondrocyte development with G1 phase advance, S phase increasing and G2-M phase transition, while GRP78 knockdown can inhibit cell growth in chondrogenesis.

### 2.6. Impact of Ad-GRP78 and Ad-siGRP78 on ER Stress-Mediated Apoptosis in Chondrocyte Differentiation

**Figure 5:**
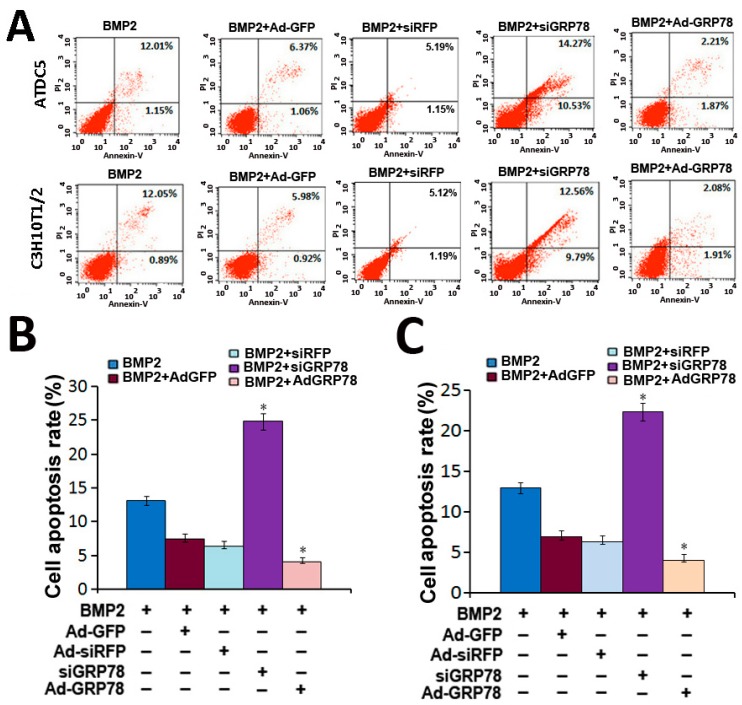
Cellular apoptosis assay with FCM. (**A**) Flow cytometry analysis with Annexin V-PI staining was performed to evaluate the percentage of apoptotic cells in BMP2 (300 ng/mL)-induced ATDC5 and C3H10T1/2 cells for three days. The percentage of apoptotic cells in the ATDC5 and C3H10T1/2 Ad-GRP78 cells groups were significantly decreased compared with that of controls. While the percentage of apoptotic cells in the ATDC5 and C3H10T1/2 Ad-siGRP78 cells groups were significantly increased compared with that of controls; (**B**) Analysis on cell apoptosis results of ATDC5 cells. Data are mean ± SD for relative apoptosis normalized to control cells for three independent experiments. Columns mean of four separate experiments; bars represent SD. * *p* < 0.05 as determined by Student’s *t*-test, versus BMP2 + Ad-siRFP and BMP2 + Ad-siGRP78 group, BMP2 + Ad-GFP and BMP2 + Ad-GRP78 group. Representative images from flow cytometry analysis are shown; (**C**) Analysis on cell apoptosis results of C3H10T1/2 cells. Data are mean ± SD for relative apoptosis normalized to control cells for three independent experiments. Columns mean of four separate experiments; bars represent SD. * *p* < 0.05 as determined by Student’s *t*-test, versus BMP2 + Ad-siRFP and BMP2 + Ad-siGRP78 group, BMP2 + Ad-GFP and BMP2 + Ad-GRP78 group. Representative images from flow cytometry analysis are shown.

In the flow cytometry assay shown in Figure 5A, the cell apoptosis rate was clearly increased after being infected with BMP2 + Ad-siGRP78 compared with BMP2 + siRFP control and BMP2 group in micromass culture of ATDC5 cells for three days. The cell apoptotic rate was 13.16%, 6.34% and 24.8% in BMP2 group, BMP2 + siRFP group and BMP2 + Ad-siGRP78 group, respectively. While the cell apoptotic rate was reduced after being infected with Ad-GRP78 compared with BMP2 + Ad-GFP control and BMP2 group. The cell apoptotic rate was 4.08% in BMP2 + Ad-GRP78 ATDC5 cells. In addition, in micromass culture of C3H10T1/2 cells, the cell apoptosis rate was also increased after being infected with BMP2 + Ad-siGRP78 group (22.35%) compared with BMP2 + siRFP control (6.31%) and BMP2 group (12.94%) in micromass culture of C3H10T1/2 cells for three days. While the cell apoptotic rate was reduced after being infected with BMP2 + Ad-GRP78 group (3.99%) compared with BMP2 group. The differences between treatment groups and BMP2 groups have statistical significance (* *p* < 0.05, Figure 5B,C). Taken together, these data demonstrate that overexpression of GRP78 can inhibit, while knockdown of GRP78 via the RNAi approach can enhance ER stress-mediated apoptosis in chondrocyte differentiation induced by BMP2.

**Figure 6:**
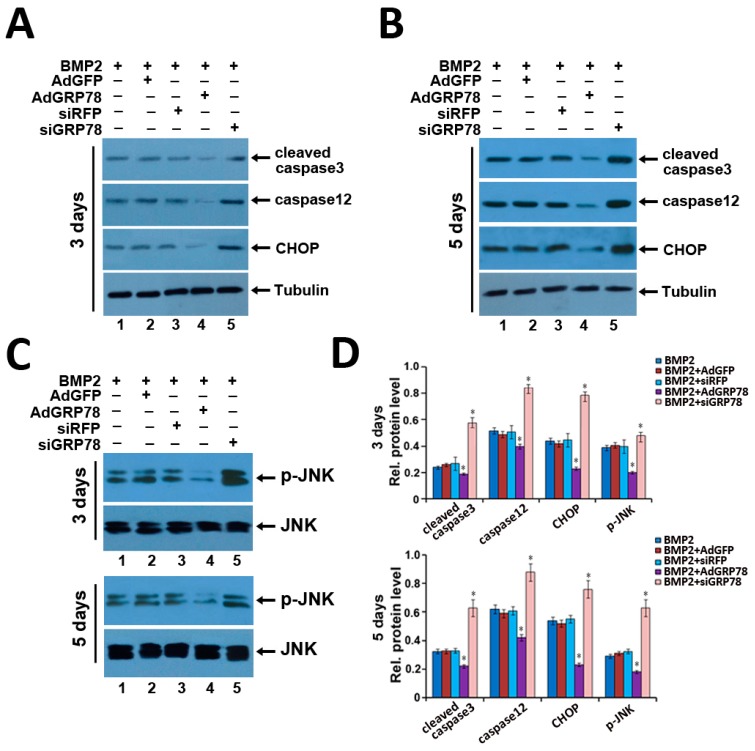
The effects of GRP78 on the expression of ER stress-associated molecules in BMP2-induced C3H10T1/2 cells for three and five days. (**A**–**C**) Expression of cleaved caspase3, CHOP, caspase12, tubulin, JNK and p-JNK in the course of chondrogenesis in a micromass culture of BMP2-induced C3H10T1/2 cells for three and five days. Whole cell lysates were prepared from C3H10T1/2 cells were treated with 300 ng/mL BMP2 for three and five days. The lysates were resolved by SDS-PAGE and then immunoblotted with antibodies against cleaved caspase3, caspase12, CHOP, JNK, p-JNK and Tubulin; (**D**) Semi-quantification of relative levels of cleaved caspase3, caspase12, CHOP and p-JNK. Levels were normalized against those of tubulin by MJ Opticon Monitor Analysis Software (Bio-Rad), data were expressed as means ± S.D. (*n* = 3). * *p* < 0.05 as determined by Student’s *t*-test, *versus* BMP2 + Ad-siRFP and BMP2 + Ad-siGRP78 group, BMP2 + Ad-GFP and BMP2 + Ad-GRP78 group.

Then, to confirm the influence of ER stress-mediated apoptosis by GRP78 in BMP2-induced C3H10T1/2 cells, the expression of ER stress-mediated apoptosis molecules such as CHOP, cleaved caspase3, caspase12 and phosphorylated JNK was detected by Western blot in C3H10T1/2 cells induced by BMP2 for three and five days. The result showed that cleaved caspase3, caspase12, CHOP and p-JNK expressions dramatically increased after being infected with Ad-siGRP78 compared with BMP2 + siRFP control and BMP2 group in C3H10T1/2 cells induced by BMP2 for three or five days. As revealed in Figure 6, Ad-siGRP78 induced a dramatic augment in active (cleaved) caspase3, caspase12, p-JNK and CHOP expressions in C3H10T1/2 with Ad-siGRP78 cells. However, cleaved caspase3, caspase12, p-JNK and CHOP expressions were obviously reduced after being infected with Ad-GRP78 compared with BMP2 + AdGFP control, and the BMP2 group in C3H10T1/2 cells induced by BMP2 for three or five days. It was demonstrated that Ad-GRP78 could inhibit the expression of ER stress-mediated apoptosis signal pathway molecules in chondrogenesis. And Ad-siGRP78 promoted the expression of ER stress-mediated apoptosis molecules in chondrocyte differentiation.

### 2.7. Ad-GRP78 Inhibition of the ER Stress-Mediated Apoptosis in Vitro and in Vivo

**Figure 7:**
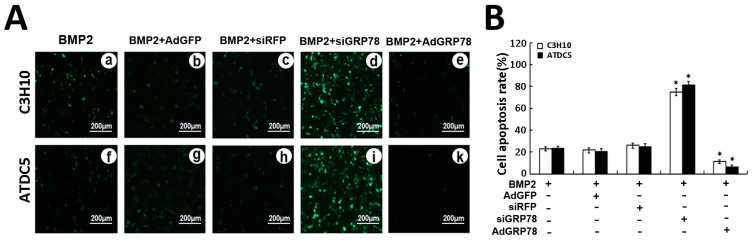
Ad-GRP78 inhibits, while siGRP78 increases, ER stress-mediated apoptosis in micromass culture of ATDC5 cells and C3H10T1/2 cells induced by BMP2. (**A**) After treatment with 300 ng/mL BMP2, BMP2 + Ad-GFP, BMP2 + Ad-GRP78, BMP2 + siRFP or BMP2 + siGRP78 in micromass culture of C3H10T1/2 cells (**a**–**e**) and ATDC5 cells (**f**–**k**), then were analyzed for apoptosis using the TUNEL staining assay. Representative photographs of TUNEL staining in cells. The FITC-labeled TUNEL-positive cells were imaged under a fluorescent microscope (Bar = 200μm). The cells with green fluorescence were recognized as apoptotic cells, and the scale bars represent 200 μm; (**B**) Analysis on cell apoptosis results. Data are mean ± SD for relative apoptosis normalized to control cells for three independent experiments. Columns mean of three separate experiments; bars represent SD. * *p* < 0.05 as determined by Student’s *t*-test, versus BMP2 + siRFP and BMP2 + siGRP78 group; BMP2 + Ad-GFP and BMP2 + Ad-GRP78 group. Representative images from TUNEL analysis are shown.

Next we compared the roles of GRP78 during chondrogenesis using ATDC5 cells and C3H10T1/2, which are capable of differentiation into various lineages, such as chondrocytes. In brief, the high-density culture system was incubated in the absence (CTR) or presence of 300 ng/mL BMP2, BMP2 + Ad-GFP,BMP2 + Ad-siRFP,BMP2 + Ad-GRP78 and BMP2 + Ad-siGRP78 for three days, TUNEL assay was undertaken to determine the effect of GRP78 on apoptosis in chondrogenesis. As shown in Figure 7A, during the BMP2-induced chondrocyte differentiation, the number of TUNEL-positive cells significantly increased in the ATDC5 BMP2 + Ad-siGRP78 cells (80.56%) compared with ATDC5 BMP2 + Ad-siRFP cells (19.52%) and BMP2 cells (21.23%), while the number of TUNEL-positive cells reduced in the ATDC5 BMP2 + Ad-GRP78 cells (5.02%).

Incidentally, in the C3H10T1/2 BMP2 + Ad-siGRP78 cells, the number of TUNEL-positive cells obviously increased (76.13%) compared with C3H10T1/2 BMP2 cells (23.02%), while the number of TUNEL-positive cells reduced in the C3H10T1/2 BMP2 + Ad-GRP78 cells (8.29%). The differences between BMP2 + Ad-GRP78, BMP2 + Ad-siGRP78, BMP2 + Ad-GFP, BMP2 + Ad-siRFP, BMP2 groups have statistical significance (* *p* < 0.05 Figure 7B). Note that GRP78 can inhibit ER stress-mediated apoptosis in chondrocyte differentiation induced by BMP2.

**Figure 8:**
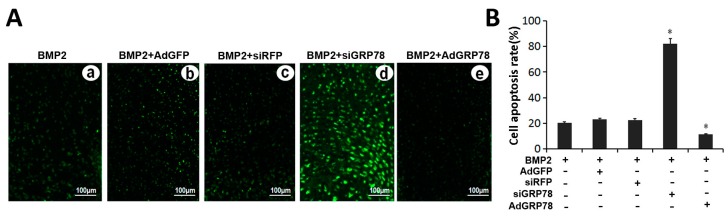
TUNEL staining in the growth plate chondrocytes *in vivo*. (**A**) Metatarsals were explanted from 15-day-old mouse embryos and cultured in the presence of conditioned medium of BMP2 (300 ng/mL) (**a**); BMP2 + Ad-GFP (**b**); BMP2 + siRFP (**c**); BMP2 + siGRP78 (**d**); BMP2 + Ad-GRP78 (**e**) for five days and then were analyzed for apoptosis using the TUNEL-staining assay. Representative photographs of TUNEL staining in cells. The FITC-labeled TUNEL positive cells were imaged under a fluorescent microscope (Bar = 100 μm). The cells with green fluorescence were recognized as apoptotic cells, and the scale bars represent 100 μm; (**B**) Analysis on cell apoptosis results. Data are mean ± SD for relative apoptosis normalized to control cells for three independent experiments. Columns mean of three separate experiments; bars represent SD. * *p* < 0.05 as determined by Student’s *t*-test, versus BMP2 + siRFP and BMP2 + siGRP78 group; BMP2 + Ad-GFP and BMP2 + Ad-GRP78 group. Representative images from TUNEL analysis are shown.

Then to verify whether GRP78 influence the growth plate chondrocytes in developing tissue, TUNEL assay was undertaken to determine the effect of GRP78 on apoptosis in chondrocyte tissue (Figure 8). The result showed that the BMP2 + Ad-siGRP78 group resulted in much more apoptotic cells compared with the BMP2 group and BMP2 + siRFP group, whereas the TUNEL-positive cells in the BMP2 + Ad-GRP78 group were obviously reduced compared with the BMP2 group and BMP2 + Ad-GFP group.

### 2.8. Different Influence of Ad-siGRP78 on the ER Stress Specific Caspase Cascade in Developing Chondrocyte Tissue at E17.5

To further understand the molecular events of the ER stress-mediated apoptosis influenced by GRP78 in chondrogenesis, the effect of GRP78 on endochondral bone formation was then studied in an ex vivo model of bone formation using cultures of 17-day-old fetal mouse metatarsals. Firstly, the metatarsals were cultured for five days in the presence of the conditioned medium obtained from BMP2 (control), BMP2 + siRFP (control) and BMP2 + siGRP78 adenovirus. At the time of explantation, these explants consisted of undifferentiated cartilage.

**Figure 9:**
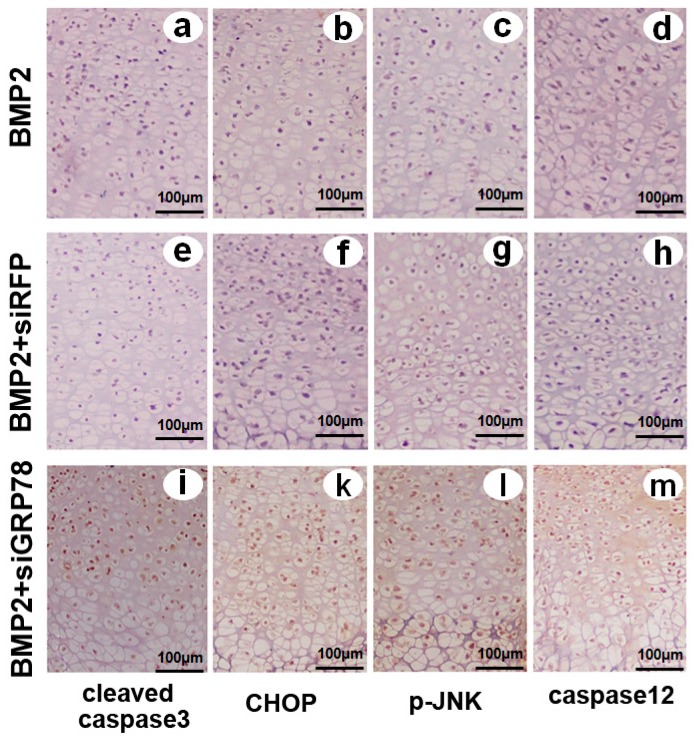
Expression of cleaved caspase3, CHOP, caspase12 and p-JNK in the growth plate chondrocytes *in vivo*. Metatarsals were explanted from 17-day-old mouse embryos and cultured in the presence of conditioned medium of BMP2 (300 ng/mL), BMP2 + siRFP, BMP2 + siGRP78 for 5 days. (**a**), (**e**) and (**i**) Immunohistochemistry staining was observed in low-power microphotograph of a section stained with anti-active caspase3 monoclonal antibody (brown) and counterstained with Mayer’s hematoxylin (blue); (**b**), (**f**) and (**k**) Immunohistochemistry staining was observed in a low-power microphotograph of a section stained with anti-CHOP monoclonal antibody (brown) and counterstained with Mayer’s hematoxylin (blue); (**c**), (**g**) and (**l**) Immunohistochemistry staining was observed in low-power microphotograph of a section stained with anti-p-JNK monoclonal antibody (brown) and counterstained with Mayer’s hematoxylin (blue); (**d**), (**h**) and (**m**) Immunohistochemistry staining was observed in low-power microphotograph of a section stained with anti-caspase12 monoclonal antibody (brown) and counterstained with Mayer’s hematoxylin (blue) and the scale bars represent 100 μm.

In a five-day culture period, these explants underwent all sequential stages of endochondral bone formation. After five days of culture, the explants were fixed, and then stained with hematoxylin and eosin stain (HE stain). Immunohistochemistry analysis of embryonic metatarsal tissues was made with anti-active caspase3, caspase12, CHOP, p-JNK monoclonal antibody, respectively. As shown in Figure 9, Ad-siGRP78 enhanced the apoptosis-related protein expression in chondrocyte differentiation, such as cleaved caspase3, caspase12, CHOP, and p-JNK. It was demonstrated that the activation of caspase3 and caspase12, phosphorylation of JNK and up-regulation of CHOP by ER stress occurred in chondrogenesis. Further, Ad-siGRP78 could improve the expression of ER stress-mediated apoptosis signal pathway molecules in chondrogenesis compared with BMP2 and BMP2 + siRFP controls.

Taken together, these data demonstrate that GRP78 inhibits ER stress-mediated apoptosis in chondrocyte differentiation induced by BMP2.

## 3. Discussion

The endoplasmic reticulum is an essential organelle for multiple cellular functions such as maintaining calcium homeostasis and the biosynthesis of proteins or lipids. Many studies have shown that factors influencing cell fate and/or differentiation are activated in ER stress. UPR is primarily a response to relieve ER stress and promote survival. In mammalian cells, the UPR plays a fundamental role in maintaining cellular homeostasis and is therefore at the center of many normal physiological responses and pathologies [19,20,21,22]. The UPR includes three molecular branches, which are IRE1α, PERK and ATF6. These three-signal pathways trigger both cell protective and cell death responses. When the severity of ER stress exceeds the capacity of the UPR to restore homeostasis, mammalian cells commit to apoptosis, which is called ER stress-induced cell apoptosis. UPR may alter apoptosis of hypertrophic chondrocytes, which is thought to be a normal process prior to the conversion to bone [23,24,25,26].

Chondrogenesis is a process that is important for the creation of chondrocytes both during embryogenesis as well as in adult life. Cartilage development and growth is controlled through the highly coordinated proliferation, apoptosis, and differentiation of growth plate chondrocytes [27,28]. Saito *et al.* [29,30] reported that treatment of wild-type primary osteoblasts with BMP2-induced ER stress, leading to an increase in ATF4 protein expression levels. During osteoblastogenesis, the expression levels of the ER stress markers, such as BiP, CHOP, ATF4, and EDEM (ER degradation enhancing α-mannosidase-like protein), were up-regulated by BMP2 stimulation. UPR is primarily a response to relieve ER stress and promote survival. UPR may alter apoptosis of hypertrophic chondrocytes, which is thought to be a normal process prior to the conversion to bone.

Some studies agree with the fact that an ER chaperone protein GRP78 serves as a master UPR regulator and plays essential roles in activating PERK, IRE1, and ATF6 in response to ER stress [20,31]. GRP78, also known as BiP, is a multi-functional protein predominantly expressed in the lumen of the ER. Typically, GRP78 acts as a major ER chaperone and a master regulator of ER stress signaling through controlling protein folding and assembly, preventing protein aggregation, and regulating signaling of the unfolded protein response [32,33,34,35]. However, whether GRP78 impact differentiation programs in chondrocytes is poorly understood; whether GRP78 participates in ER stress-mediated apoptosis in the process of chondrocyte differentiation, and the mechanism on how to regulate ER stress-mediated apoptosis in chondrogenesis remain unknown. Our current study focuses on the role of GRP78 in ER stress-mediated apoptosis during the process of chondrogenesis as well as the molecular mechanism involved.

Herein, we constructed Ad-GRP78 and Ad-GRP78 siRNA adenovirus that infected into ATDC5 cells and C3H10T1/2 cells. The results showed that Ad-GRP78 increased, while Ad-GRP78 siRNA inhibited the expression of GRP78 in C3H10T1/2 cells (Figure 1) and in metatarsals from 15.5-day-old, 18.5-day-old mouse embryos and newborn mice. Besides, GRP78 is detected and prominently expressed throughout the growth plate chondrocytes (Figure 2). Then, we induced C3H10T1/2 and ATDC5 cells with BMP2 for induction of chondrocyte differentiation. The result showed that GRP78 is able to up-regulate XBP1S gene expression, and GRP78 is required for XBP1S expression in C3H10T1/2 cells induced by BMP2. Then, it was detected that GRP78 can enhance the level of IRE1α-spliced XBP1S protein in chondrogenesis induced by BMP2. IRE1α and GRP78 can synergistically regulate endogenous XBP1S gene expression in chondrogenesis. GRP78 decreases the expression of XBP1S and reduces the BMP2-induced IRE1α kinetic activity in chondrogenesis (Figure 3). It was reported that GRP78 is a peptide-dependent ATPase that binds transiently to newly synthesized proteins translocated into the ER and more permanently to under glycosylated, misfolded, or unassembled proteins. Under non-stressed conditions, GRP78 also binds to the luminal domains of IRE1, PERK, and ATF6 to maintain them within the ER. Upon accumulation of unfolded proteins, GRP78 is released from IRE1 and PERK to permit their spontaneous: dimerization/oligomerization, trans-autophosphorylation, and subsequent activation. Therefore, in BMP2-induced chondrocyte differentiation, IRE1α kinetic activity was activated; GRP78 reduced the BMP2-induced IRE1α kinetic activity and IRE1α signaling.

Then we determined whether and how GRP78 influences ER stress-mediated apoptosis during chondrocyte differentiation. We detected and found that GRP78 can influence cell cycle distribution in chondrogenesis. Overexpression of GRP78 enhanced, while GRP78 knockdown inhibited, the S phase cells in chondrocyte differentiation. It was demonstrated that overexpressed GRP78 can obviously improve cell proliferation activity in chondrocyte development with G1 phase advance, S phase increasing and G2-M phase transition, while GRP78 knockdown can inhibit cell growth in chondrogenesis (Figure 4). Caspases, a family of cysteine proteases, act as common death effector molecules in various forms of apoptosis [36,37]. During apoptosis, caspases are activated by different mechanisms. In addition, upon ER stress, activation of UPR sensor PERK, IRE1, or ATF6 leads to transcriptional activation of CHOP/GADD153, a bZIP transcription factor that potentiates apoptosis. These findings support the notion that ER stress leads to several redundant pathways for caspase activation [38,39,40]. Then, we determined whether and how GRP78 influence ER stress mediated apoptosis during chondrocyte differentiation. Both FCM and Western blot results showed that overexpression of GRP78 inhibits ER stress-mediated apoptosis in chondrocyte differentiation induced by BMP2; whereas knockdown of GRP78 via RNAi approach enhances the expression of ER stress-mediated apoptosis signal pathway molecules under the procedure of chondrogenesis. Ad-GRP78 can reduce ER stress-mediated apoptosis in chondrocyte differentiation induced by BMP2, as assayed by cleaved caspase3, caspase12, CHOP and p-JNK expression in the course of chondrocyte differentiation (Figure 5 and Figure 6). Caspase12 is an ER-associated proximal effector in the caspase activation cascade, and cells lacking this enzyme are partially resistant to inducers of ER stress. Caspase12 can activate caspase9, which in turn activates caspase3. Identification of ER stress as a trigger was made possible by detection of caspase12 activation because its activation is nearly synonymous with generation of ER stress [41,42,43]. Furthermore, TUNEL assay results showed that Ad-GRP78 can inhibit, while knockdown of ATF6 via RNAi approach can improve, ER stress-mediated apoptosis in chondrogenesis (Figure 7). On the other hand, Ad-siGRP78 enhanced ER stress-mediated apoptosis in cartilage tissue induced by BMP2 with tissue TUNEL assay (Figure 8) and immunohistochemistry (Figure 9). It was demonstrated that Ad-siGRP78 activation of the ER stress-specific caspase cascade in developing chondrocyte tissue.

In conclusion, our work supports that GRP78 is a negative regulator of ER stress-mediated apoptosis in chondrocyte differentiation. The GRP78 inhibition of apoptosis could influence the differentiation of the chondrocytes, and the crosstalk mechanism between apoptosis and differentiation needs further investigation. This study provides novel insights into the role of GRP78 in regulating the ER stress and ER stress-mediated apoptosis in chondrogenesis. The inhibition of GRP78 in apoptosis allows the cell to adapt to physiological changes in response to pathological conditions that are associated with ER stress. In addition, research so far has identified many candidates involved in orchestrating the switch from the protective UPR signaling to pro-apoptotic signaling. New insights into the mechanistic basis of stress responses will open new perspectives for the development of molecular-targeted treatment approaches and thus have a great potential for the treatment of cartilage disorders and arthritic conditions.

## 4. Experimental Section

### 4.1. Ethics statement

With the approval of the Chongqing Medical University Institutional Animal Care and Use Committee (Permit Number: SYXK 2007-0001, SCXK 2007-0002), all mice were housed under controlled temperatures in a 12 h light/dark cycle with easy access to food and water. This study was carried out in strict accordance with the recommendations in the Guide for the Care and Use of Laboratory Animals of the National Science Foundation of China. The protocol was approved by the Committee on the Ethics of Animal Experiments of Chongqing Medical University. All surgery was performed under sodium pentobarbital anesthesia, and all efforts were made to minimize suffering.

### 4.2. Adenoviruses Production & Titration

The AdEasy adenoviral vector system (Invitrogen, Carlsbad, CA, USA) was used to construct an adenovirus expressing GRP78 (Ad-GRP78). Briefly, GRP78 cDNA was inserted into the BamHI and HindIII sites in pAdTrack-cytomegalovirus (CMV) vector. The corresponding segments *GRP78* were amplified using PCR with the following primers: Sense: 5′-GGATCCATGAAGCTCTCCCTGGTG-3′, Antisense: 5′-AAGCTTGGGCAACTCATCTTTTTCTG-3′. The enzyme sites in the primers are underlined. PCR products were inserted into the pAdTrack vector. Then the predigested recombinant adenovirus DNA was transfected into human embryonic kidney 293 cells. After collecting the medium supernatant that contains recombinant adenovirus, multiplicity of infection (MOI) for the recombinant adenovirus was determined according to the standard protocol [44,45]. The expression of recombinant virus in infected GRP78 cells was tested by Western blotting with specific antibodies.

To generate adenovirus *GRP78* siRNA, the siRNA sequence corresponding to the *GRP78* gene (5′-CTGTGGCTGGACTGCCTGTTT-3′) was cloned into a pSES-HUS vector (siRNA adenoviral shuttle vector). Briefly, equimolar amounts of complementary sense and antisense strands were separately mixed, annealed, and slowly cooled to 10 °C in a 50 μL reaction buffer. The annealed oligonucleotides were inserted into the SfiI sites of pSES-HUS vector. Adenovirus GRP78 siRNA, and adenovirus encoding GRP78 were constructed, respectively, using methods described previously [46,47]. All constructs were verified by nucleic acid sequencing; subsequent analysis was performed using BLAST software [48].

### 4.3. Cell Culture

The micromass culture was performed as described previously. The ATDC5 cells and C3H10T1/2 cells were briefly trypsinzed and then resuspended in DMEM with 10% FBS at a concentration of 10^6^ cells per mL, and six drops of 100 mL of cells were placed in a 60 mm tissue culture dish (Becton Dickinson, New York, NY, USA). After 2 h of incubation at 37 °C, 1 mL of DMEM containing 10% FBS and BMP2 protein (300 ng/mL) was added. The media was replaced approximately every 2–3 days. To test the effect of overexpression of GRP78 on chondrogenesis, ATDC5 cells were infected with Ad-GRP78 expression adenovirus or control GFP adenovirus before micromass culture.

### 4.4. RNA Preparation and Reverse Transcription (RT)-PCR

Total RNA was prepared from ATDC5 cells; control lines were cultured in tissue culture dishes in Dulbecco’s Modified Eagle’s Medium (DMEM) supplemented with 10% heat inactivated fetal calf serum and antibiotics using the QIAGEN (QIAGEN Inc., Hilden, Germany) RNeasy mini kit and reverse transcribed using oligo (dT) primers with the SuperScript pream purification system (Invitrogen) following the manufacturer’s instructions. The following sequence-specific primers were synthesized: sense, 5′-TTTGAGCACATGCTTCGCTG-3′; antisense, 5′-AAGCTTGGAGGGCGTCTGGAGTCAC-3′ for GRP78. The following pair of oligonucleotides was used as internal controls: 5′-ACCACAGTCCATGCCATCAC-3′ and 5′-TCCACCACCCTGTTGCTGTA-3′ for GAPDH. PCRs were performed for 35 cycles (94 °C, 1 min, 58 °C, 2 min and 72 °C, 1 min) with a final ligation for 10 min at 72 °C. GAPDH was also amplified and employed as an internal control for 35 cycles (94 °C, 1 min, 55 °C, 1 min and 72 °C, 1 min). PCR products were visualized on 1% agarose gels containing 0.1 mg/mL ethidium bromide using ultraviolet light. The identity of each targeted PCR amplification product was confirmed by DNA sequence analysis of gel-purified bands (QIAGEN Inc., Hilden, Germany).

### 4.5. Immunoblotting Analysis

To examine the expressions of cleaved caspase3, caspase12, CHOP, tubulin, JNK (c-Jun N-terminal kinase) and p-JNK protein in the course of chondrogenesis, total cell extracts prepared from micromass cultures of ATDC5 cells in the presence of 300 ng/mL recombinant BMP2 protein were mixed with 5× sample buffer (312.5 mM Tris-HCl (pH 6.8), 5% β-mercaptoethanol, 10% SDS, 0.5% bromophenol blue, 50% glycerol). Proteins were resolved on a 10% SDS-polyacrylamide gel and electroblotted onto a nitrocellulose membrane. After blocking in 10% nonfat dry milk in Tris buffer saline Tween 20 (10 mM Tris-HCl (pH 8.0), 150 mM NaCl, 0.5% Tween 20), blots were incubated with either mouse monoclonal anti-IRE1α antibody and anti-CHOP antibody (BioLegend, San Diego, CA, USA.) (diluted 1:1000) or rabbit polyclonal anti-cleaved caspase3 antibody and anti-p-JNK antibody (Santa Cruz Biotechnology, 1:1000) for 1 h. After washing, the respective secondary antibody (HRP-conjugated anti-mouse immunoglobulin or HRP-conjugated anti-rabbit immunoglobulin, (Sigma, St. Louis, MO, USA, both 1:5000 dilutions) was added, and bound antibody was visualized using an enhanced chemiluminescence system (GE Amersham Biosciences, Amersham, Buckinghamshire, UK).

### 4.6. Quantitative Real-Time PCR

Real-time PCR with SYBR Green chemistry was performed to check the mRNA level of XBP1S after being infected with Ad-siGRP78 or Ad-GRP78 into C3H10T1/2 cells, respectively, using the ABI prism 7500 PCR amplification system (PE Biosystems, Foster City, CA, USA). The sequences of XBP1S-specific primers are 5′-ATGGTGGTGGTGGCAGCCGC-3′ and 5′-GACACTAATCAGCTGG GGAAAGAG-3′. The sequences of human GAPDH-specific primers are 5′-TGAAGGTCGGAGTC AACGGATTTGGT-3′ and 5′-CATGTGGGCCATGAGGTCCACCAC-3′. A reaction mixture containing the master mixture with SYBR Green fluorescent dye (Roche Diagnostics, Basel, Switzerland) and the selective primers were added to a 96-well plate, together with 2 μL of cDNA template, for a final reaction volume of 20 μL per well, and run for an initial step at 95 °C for 10 min, followed by 40 cycles of amplification at 95 °C for 15 s and 60 °C for 60 s. All the data were collected during the extension step and expressed as arbitrary fluorescence units per cycle. A melting curve was obtained at the end of the PCR reaction to verify that only one product was produced.

### 4.7. Apoptosis Analysis by Flow Cytometry (FCM)

The ATDC5 cells and C3H10T1/2 cells were infected with Ad-GRP78, Ad-siGRP78 respectively, then the cell cycle analysis and cell apoptotic rate were determined by FCM analysis. The percentage of cells with the sub-G1 DNA content reflects the apoptotic rate of the cell population. At 48 h post-transfection, the culture media were collected. The cells in 35 mm dishes were trypsinized and fixed with 70% ethanol for more than 1 h. Cells were pelleted and washed with PBS plus 20 mM EDTA. After being incubated with RNase (1 mg/mL, QIAGEN Inc., Hilden, Germany) at 37 °C for 1 h, cells were stained with propidium iodide (30 mg/mL, Sigma) and analyzed by flow cytometry to measure cell cycle distribution and cell apoptosis rate (Becton Dickinson FACS Calibur, New York, NY, USA). The experiments were performed in triplicate.

### 4.8. TUNEL Assay

Apoptotic cells were detected by terminal deoxynucleotidyl-transferase-mediated dUTP-biotin nick end-labeling staining using the Dead End TM Fluorometric TUNEL system (Promega, Madison, WI, USA). Cells were processed according to the manufacturer’s recommended protocol. Briefly, the cells were fixed with 4% paraformaldehyde phosphate buffer saline, rinsed with PBS, treated with 0.1% Triton X-100 for 2 min on ice, and incubated with TUNEL for 1 h at 37 °C. The FITC-labeled TUNEL-positive cells were imaged under a fluorescent microscope. The cells with green fluorescence were recognized as apoptotic cells.

### 4.9. Culture of Fetal Mouse Bone Explants and Immunohistochemistry

Fetal mouse metatarsals were dissected from fetal C57BL/6J mice (E17.5) and cultured in DMEM (Gibco) containing 1% heat-inactivated fetal calf serum (Invitrogen) and 100U penicillin-streptomycin per milliliter in the various stimuli (BMP2, BMP2 + Ad-GRP78). After 5 days of culture, for histological examination, every group tissue samples make five-micrometer-thick, formalin-fixed paraffin sections, then sections were deparaffinized, dehydrated and placed in Tris buffer (10 mM Tris-HCl (pH 8.0), 150 mM NaCl). Serum block was applied for 30 min at room temperature before incubation of the primary antibody. Affinity-purified polyclonal anti-caspase3, anti-CHOP, and anti-p-JNK were diluted at 1:100 and sections were incubated at room temperature for 2 h respectively. For detection, biotinylated secondary antibody and horseradish peroxidase (HRP)-streptavidin complex (Santa Cruz Biotechnology, Delaware Ave, Santa Cruz, CA, USA) were used. A total of 0.5 mg/mL 3,3′-diaminobenzidine (DAB) in 50 mM Tris-HCl substrate (Sigma) was used for visualization, and sections were then counterstained with Mayer’s hematoxylin.

### 4.10. Statistical Test

The statistical analysis was performed with SPSS 10.0.1 software for Windows. Data were expressed as mean ± SD from at least three independent experiments. Data for multiple variable comparisons were analyzed by one-way analysis of variance (ANOVA). *p* values <0.05 were deemed statistically significant.
