# Correction: Xiong, Z., *et al*. Different Roles of GRP78 on Cell Proliferation and Apoptosis in Cartilage Development. *Int. J. Mol. Sci.* 2015, *16*, 21153–21176

**DOI:** 10.3390/ijms161226222

**Published:** 2015-12-17

**Authors:** Zhangyuan Xiong, Rong Jiang, Xiangzhu Li, Yanna Liu, Fengjin Guo

**Affiliations:** 1 Department of Cell Biology and Genetics, Core Facility of Development Biology, Chongqing Medical University, Chongqing 400016, China; zyxiong38@sina.com (Z.X.); lixzh11@sina.com (X.L.); yanlii@126.com (Y.L.); 2 Laboratory of Stem Cells and Tissue Engineering, Chongqing Medical University, Chongqing 400016, China; Rongjiang86@sohu.com

The authors wish to replace Figure 4A on Page 21161 of their paper published in *IJMS* [1]. There was an error in the representative image of BMP2 + Ad-GRP78 group of C3H10T1/2 in the Figure 4A. The images related to ATDC5 was repeated to show the BMP2 + Ad-GRP78 effects on C3H10T1/2. Please see the corrected Figure 4 here. The authors apologize for any inconvenience. The manuscript will be updated and the original will remain online at the article webpage.

**Figure 4:**
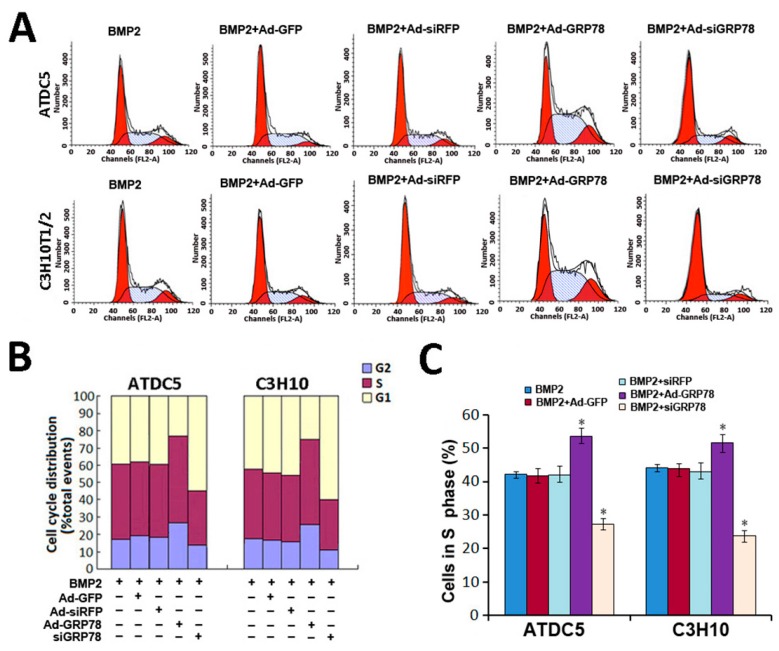
Cellular proliferation analysis by FCM. (**A**) Flow cytometry images with propidium iodide staining and analysis on cell cycle distribution. Micromass culture of ATDC5 cells and C3H10T1/2 were treated with BMP2 (300 ng/mL) / BMP2 + Ad-GFP/BMP2 + Ad-siRFP / BMP2 + Ad-GRP78 / BMP2 + Ad-siGRP78. Flow cytometry analysis showed that the percentage of the BMP2 + Ad-GRP78 ATDC5 cells in S phase were increased significantly compared to those in BMP2 controls, whereas the percentage of the BMP2 + Ad-siGRP78 ATDC5 cells in S phase were dramatically decreased compared with BMP2 control. The result of C3H10T1/2 is the same. Experiments were repeated three times, and samples were analyzed by Student’s *t*-test and statistical significance with *p* < 0.05. Representative images were shown; (**B**) Flow cytometry assay on the percentages of the ATDC5 and C3H10T1/2 cells in G2/M phase after treatment with BMP2 (300 ng/mL)/BMP2 + Ad-GFP / BMP2 + Ad-siRFP / BMP2 + Ad-GRP78 / BMP2 + Ad-siGRP78; (**C**) Flow cytometry analysis showed that the percentages of the ATDC5 and C3H10T1/2 Ad-GRP78 cells in S phase were increased significantly, whereas the percentages of the ATDC5 and C3H10T1/2 Ad-siGRP78 cells in S phase were decreased compared with those in their controls. * *p* < 0.05 compared with control.
